# Supplementary material for: Tracking down the White Plague. Chapter two: The role of endocranial abnormal blood vessel impressions and periosteal appositions in the paleopathological diagnosis of tuberculous meningitis
Source: PLoS One. 2020 Sep 1;15(9):e0238444. doi: 10.1371/journal.pone.0238444 (PMC7462305; doi:10.1371/journal.pone.0238444)
Supplement: S1 Table — (MR = morgue record; DC1 = death certificate primary; DC2 = death certificate secondary; DC3 = death certificate tertiary; c. = circa; F = female; M = male; TB = tuberculosis; ABVIs = abnormal blood vessel impressions; PAs = periosteal appositions; + = exhibiting ABVIs/PAs;– = not exhibiting ABVIs/PAs). (PDF) [file pone.0238444.s001.pdf]

**S1 Table: Basic biographic data of individuals in the TB group (N=234).**
**(MR = morgue record; DC1 = death certificate primary; DC2 = death certificate secondary; DC3 = death certificate tertiary; c. = circa; F = female; M = male; TB = tuberculosis; ABVIs = abnormal blood vessel impressions; PAs = periosteal appositions; + = exhibiting ABVIs/PAs; – = not exhibiting ABVIs/PAs)**

| No. | Terry No. | Age at death | Sex | Cause of death | | | | Exhibiting ABVIs or not | Exhibiting PAs or not |
|---|---|---|---|---|---|---|---|---|---|
| | | | | MR | DC1 | DC2 | DC3 | | |
| 1 | 13R | 28 years | M | Pulmonary TB | – | Infection of the right toe | – | + | – |
| 2 | 23R | 49 years | F | Pulmonary TB | – | – | – | + | – |
| 3 | 30R | 26 years | M | TB | – | TB meningitis | – | – | – |
| 4 | 35R | 60 years | M | Pulmonary TB | – | – | – | – | – |
| 5 | 39 | 22 years | F | TB, pneumonia | Pneumonia | – | – | – | – |
| 6 | 54 | 36 years | M | TB, diarrhoea | Pulmonary TB | – | – | – | – |
| 7 | 84 | c. 24 years | M | Pulmonary TB | Pulmonary TB | – | – | + | – |
| 8 | 87R | 73 years | F | Lues | Lues | Suspected TB | – | – | – |
| 9 | 89R | c. 32 years | M | TB | – | – | – | – | – |
| 10 | 90 | 18 years | M | – | Ichthyosis | Pulmonary TB | – | – | – |
| 11 | 91R | c. 65 years | F | Pulmonary condition | Pulmonary TB | – | – | – | – |
| 12 | 95 | c. 35 years | F | Pulmonary TB | – | – | – | + | – |

| No. | Terry No. | Age at death | Sex | Cause of death | | | | Exhibiting ABVIs or not | Exhibiting PAs or not |
|---|---|---|---|---|---|---|---|---|---|
| | | | | MR | DC1 | DC2 | DC3 | | |
| 13 | 95R | c. 30 years | M | Pulmonary TB | – | – | – | – | – |
| 14 | 103R | c. 74 years | F | – | Emphysema | TB | – | – | – |
| 15 | 114 | 43 years | F | TB | – | – | – | – | – |
| 16 | 128 | 21 years | M | TB | Pulmonary TB | – | – | + | + |
| 17 | 129 | 18 years | M | TB spondylitis | – | – | – | – | – |
| 18 | 130 | 58 years | M | Pellagra | Pulmonary TB | – | Inanition | – | – |
| 19 | 138 | 49 years | M | Pulmonary phthisis | Pulmonary TB | – | – | – | – |
| 20 | 139 | 33 years | F | TB | Pulmonary TB | – | – | – | – |
| 21 | 145R | 31 years | M | Pulmonary TB | – | – | – | + | – |
| 22 | 146R | 30 years | F | Pulmonary TB | Pulmonary TB | – | Nephritis | – | – |
| 23 | 158R | c. 32 years | F | TB | Pulmonary TB | – | – | + | – |
| 24 | 182 | 43 years | M | Pulmonary TB | Pulmonary TB | – | – | – | – |
| 25 | 194 | 73 years | M | Pulmonary TB | – | – | – | – | – |
| 26 | 204 | 24 years | M | Pulmonary TB | – | – | – | + | + |

| No. | Terry No. | Age at death | Sex | Cause of death | | | | Exhibiting ABVIs or not | Exhibiting PAs or not |
|---|---|---|---|---|---|---|---|---|---|
| | | | | MR | DC1 | DC2 | DC3 | | |
| 27 | 205 | 42 years | M | Pulmonary TB | Pulmonary TB | – | – | – | – |
| 28 | 207 | 45 years | M | Pulmonary TB | – | – | – | – | – |
| 29 | 220 | c. 56 years | M | Pulmonary TB | – | – | – | + | – |
| 30 | 222 | 20 years | M | Pulmonary TB | Pulmonary TB | – | – | – | – |
| 31 | 230 | 37 years | M | Pulmonary TB | – | – | – | – | – |
| 32 | 232R | c. 35 years | F | Pulmonary TB | – | – | – | – | – |
| 33 | 235 | 29 years | M | – | Miliary TB | – | – | + | – |
| 34 | 248R | 70 years | F | Possible TB | Pneumonia | – | – | – | – |
| 35 | 250 | 52 years | M | Laparotomy, stomach carcinoma | TB peritonitis | – | – | – | – |
| 36 | 251 | 34 years | M | TB | Pulmonary TB | – | – | – | – |
| 37 | 254 | 21 years | M | Pulmonary TB | – | – | – | + | + |
| 38 | 255 | 22 years | F | Pulmonary TB | – | – | – | + | – |
| 39 | 264 | c. 67 years | M | TB, pneumonia | – | – | – | – | – |
| 40 | 265 | 32 years | M | TB | – | – | – | + | – |

| No. | Terry No. | Age at death | Sex | Cause of death | | | | Exhibiting ABVIs or not | Exhibiting PAs or not |
|---|---|---|---|---|---|---|---|---|---|
| | | | | MR | DC1 | DC2 | DC3 | | |
| 41 | 267 | c. 42 years | M | Pulmonary TB | – | – | – | – | – |
| 42 | 269 | 20 years | M | TB | – | – | – | – | – |
| 43 | 270 | 55 years | M | TB | – | – | – | – | – |
| 44 | 279 | 34 years | M | Pulmonary TB | – | – | – | + | – |
| 45 | 280 | 24 years | F | TB | – | – | – | + | + |
| 46 | 282 | 57 years | M | Myocarditis | Pulmonary TB | Myocarditis | – | – | – |
| 47 | 283R | 40 years | M | TB, haemorrhage | Pulmonary TB | – | – | – | – |
| 48 | 284 | c. 67 years | M | TB | – | – | – | – | – |
| 49 | 304 | 20 years | F | – | Pulmonary TB | – | – | + | + |
| 50 | 306 | 18 years | F | Pulmonary TB | – | – | – | + | + |
| 51 | 309 | 45 years | M | Pulmonary TB | Pulmonary TB | TB spondylitis | Mastoiditis, sinusitis | – | – |
| 52 | 318 | 45 years | M | Pulmonary TB, myocarditis | Pulmonary TB | – | – | – | – |
| 53 | 328R | 65 years | F | Possible TB, pleurisy | Pulmonary carcinoma | Undetermined | – | – | – |
| 54 | 329 | 18 years | M | Pulmonary TB, syphilis | Pulmonary TB | Syphilis | – | + | – |

| No. | Terry No. | Age at death | Sex | Cause of death | | | | Exhibiting ABVIs or not | Exhibiting PAs or not |
|---|---|---|---|---|---|---|---|---|---|
| | | | | MR | DC1 | DC2 | DC3 | | |
| 55 | 341 | 38 years | F | Pulmonary TB | – | – | – | – | – |
| 56 | 353 | c. 37 years | M | – | TB peritonitis | – | – | + | – |
| 57 | 358R | c. 36 years | M | Pulmonary TB | – | – | – | + | – |
| 58 | 382R | 26 years | M | Pulmonary TB | – | – | – | – | – |
| 59 | 385 | 21 years | M | – | Pulmonary TB | – | – | – | + |
| 60 | 386R | 66 years | M | Pulmonary TB | – | – | – | – | – |
| 61 | 400 | c. 44 years | M | TB | – | – | – | – | – |
| 62 | 402 | c. 50 years | M | TB | – | – | – | – | – |
| 63 | 410R | 35 years | M | – | Pulmonary TB | – | – | – | – |
| 64 | 423 | c. 24 years | M | TB, pericarditis | – | – | – | – | + |
| 65 | 424 | c. 30 years | M | TB, silicosis | – | – | – | – | – |
| 66 | 426R | 70 years | M | Cardiorenal disease | Arteriosclerosis | Pulmonary TB | *Diabetes mellitus* | – | – |
| 67 | 432 | 67 years | M | Destructive nasal lesion | TB abscess | Pulmonary TB | – | – | – |
| 68 | 444 | c. 26 years | M | TB | Pulmonary TB | – | – | – | – |

| No. | Terry No. | Age at death | Sex | Cause of death | | | | Exhibiting ABVIs or not | Exhibiting PAs or not |
|---|---|---|---|---|---|---|---|---|---|
| | | | | MR | DC1 | DC2 | DC3 | | |
| 69 | 466 | 31 years | M | Pulmonary TB | – | – | – | – | – |
| 70 | 468 | 23 years | M | Pulmonary TB, TB spondylitis | – | – | – | – | – |
| 71 | 490 | c. 38 years | M | TB | – | Hepatic cirrhosis | – | – | – |
| 72 | 504 | c. 41 years | M | TB, bronchitis | Pulmonary TB | – | – | – | – |
| 73 | 522 | 30 years | M | Pulmonary TB | – | – | – | + | + |
| 74 | 523 | 47 years | M | Pulmonary TB | – | – | – | – | – |
| 75 | 537 | c. 48 years | M | Possible malaria | TB meningitis | – | – | – | – |
| 76 | 541 | 28 years | F | Pulmonary TB | – | – | – | – | – |
| 77 | 549 | 33 years | M | Myocarditis | Pulmonary TB | – | – | – | – |
| 78 | 555 | 56 years | M | Pulmonary TB | – | – | – | – | – |
| 79 | 562 | 17 years | F | Pulmonary TB | – | – | – | – | – |
| 80 | 565 | c. 32 years | M | Pulmonary TB | – | – | – | – | – |
| 81 | 566 | 40 years | M | Empyema | Empyema | Possible TB | – | – | – |
| 82 | 568 | 28 years | F | Pulmonary TB | – | – | – | + | + |

| No. | Terry No. | Age at death | Sex | Cause of death | | | | Exhibiting ABVIs or not | Exhibiting PAs or not |
|---|---|---|---|---|---|---|---|---|---|
| | | | | MR | DC1 | DC2 | DC3 | | |
| 83 | 571 | 31 years | M | TB | – | – | – | + | – |
| 84 | 572 | 60 years | M | Pulmonary TB | – | – | – | – | – |
| 85 | 575 | c. 47 years | M | Pulmonary TB | – | – | – | – | – |
| 86 | 583 | 24 years | F | Pulmonary TB | – | – | – | – | – |
| 87 | 585 | 64 years | M | Pulmonary TB | – | – | – | – | – |
| 88 | 592 | 25 years | M | Pulmonary TB | – | – | – | – | – |
| 89 | 595 | 25 years | M | Pulmonary TB | – | – | – | – | – |
| 90 | 620 | c. 55 years | M | Pulmonary TB | – | Myocarditis | – | – | – |
| 91 | 621R | 29 years | M | Pulmonary TB | Pulmonary TB | TB emphysema | – | + | + |
| 92 | 626R | c. 56 years | F | Pulmonary TB | – | – | – | – | – |
| 93 | 664 | c. 48 years | M | Pulmonary TB | Pulmonary TB | Pneumonia | – | – | – |
| 94 | 669R | 72 years | M | Heart disease | Pulmonary TB | Arteriosclerosis | Heart disease | – | – |
| 95 | 679 | 33 years | F | TB | – | – | – | – | – |
| 96 | 680 | 30 years | F | Pulmonary TB | Pulmonary TB | TB peritonitis | – | – | – |

| No. | Terry No. | Age at death | Sex | Cause of death | | | | Exhibiting ABVIs or not | Exhibiting PAs or not |
|---|---|---|---|---|---|---|---|---|---|
| | | | | MR | DC1 | DC2 | DC3 | | |
| 97 | 728R | 76 years | F | Pulmonary TB, senility | – | – | – | – | – |
| 98 | 739 | 40 years | M | TB, *diabetes mellitus* | Pulmonary TB | *Diabetes mellitus* | – | + | – |
| 99 | 752 | 71 years | M | TB, pneumonia | Pneumoconiosis | Myocarditis | Arteriosclerosis | – | – |
| 100 | 757 | 59 years | M | Pulmonary TB | – | – | – | – | – |
| 101 | 761 | c. 81 years | F | Pulmonary TB | Pulmonary TB | Myocarditis | – | – | – |
| 102 | 771 | c. 49 years | M | Pulmonary TB | – | – | – | – | – |
| 103 | 776 | c. 40 years | M | Pulmonary TB | – | – | – | – | – |
| 104 | 786 | 65 years | M | Pulmonary TB | – | – | – | – | – |
| 105 | 799 | 38 years | M | TB | – | – | – | – | – |
| 106 | 820R | 52 years | M | Pulmonary TB | – | – | – | – | – |
| 107 | 822 | 16 years | F | Pulmonary TB | – | – | – | + | – |
| 108 | 828 | 46 years | M | TB | – | – | – | – | – |
| 109 | 844 | 26 years | F | Pulmonary TB | – | – | – | – | – |
| 110 | 846 | 48 years | M | Pulmonary TB | – | – | – | – | + |

| No. | Terry No. | Age at death | Sex | Cause of death | | | | Exhibiting ABVIs or not | Exhibiting PAs or not |
|---|---|---|---|---|---|---|---|---|---|
| | | | | MR | DC1 | DC2 | DC3 | | |
| 111 | 876 | c. 22 years | M | Pulmonary TB | – | – | – | – | – |
| 112 | 892 | c. 59 years | M | Heart disease | Emphysema | Pulmonary TB | – | – | – |
| 113 | 895 | 47 years | M | TB | – | – | – | – | – |
| 114 | 896RR | 26 years | F | Pulmonary TB | – | – | – | + | – |
| 115 | 897 | 43 years | M | Pulmonary TB | Pulmonary TB | Myocarditis | – | – | + |
| 116 | 902 | c. 36 years | M | Pulmonary TB | – | – | – | + | – |
| 117 | 907 | 41 years | F | Pulmonary TB | – | – | – | – | – |
| 118 | 914 | 30 years | M | TB | – | – | – | – | – |
| 119 | 915 | 27 years | M | Pulmonary TB | – | – | – | – | + |
| 120 | 932 | 27 years | M | Pulmonary TB | – | – | – | + | + |
| 121 | 933R | 40 years | M | Probable TB, ascites | TB peritonitis | – | – | + | – |
| 122 | 936 | 56 years | M | Pulmonary TB | – | – | – | – | – |
| 123 | 950 | 59 years | M | Pulmonary TB | – | – | – | – | – |
| 124 | 952 | 41 years | F | TB | – | – | – | – | – |

| No. | Terry No. | Age at death | Sex | Cause of death | | | | Exhibiting ABVIs or not | Exhibiting PAs or not |
|---|---|---|---|---|---|---|---|---|---|
| | | | | MR | DC1 | DC2 | DC3 | | |
| 125 | 955 | 26 years | M | Pulmonary TB | – | – | – | + | + |
| 126 | 957 | 56 years | F | Chest contusion | Pulmonary TB | – | – | – | – |
| 127 | 975 | 60 years | M | TB, cardiac decompensation | Myocarditis | – | – | – | – |
| 128 | 987 | 23 years | M | Pulmonary TB | – | – | – | + | + |
| 129 | 1002 | 50 years | M | Pulmonary TB | – | – | – | – | – |
| 130 | 1005 | 52 years | M | Pulmonary TB | – | – | – | – | – |
| 131 | 1013 | c. 30 years | M | Pulmonary TB | – | – | – | – | – |
| 132 | 1018 | 30 years | M | Pulmonary TB | – | – | – | – | – |
| 133 | 1020 | 24 years | M | Pulmonary TB | – | – | – | + | – |
| 134 | 1027 | c. 41 years | F | Pulmonary TB | – | – | – | + | + |
| 135 | 1030 | 62 years | M | – | Pulmonary TB | – | – | + | – |
| 136 | 1031 | 27 years | M | Pneumonia | Pulmonary TB | – | – | – | – |
| 137 | 1033 | 26 years | M | Pulmonary TB | – | – | – | + | + |
| 138 | 1034 | 48 years | F | Pulmonary TB | TB nephritis | – | – | – | + |

| No. | Terry No. | Age at death | Sex | Cause of death | | | | Exhibiting ABVIs or not | Exhibiting PAs or not |
|---|---|---|---|---|---|---|---|---|---|
| | | | | MR | DC1 | DC2 | DC3 | | |
| 139 | 1036 | 38 years | M | Asthma, heart disease | Pulmonary TB | – | – | – | – |
| 140 | 1043 | 46 years | M | Pulmonary TB | – | – | – | – | – |
| 141 | 1047 | c. 61 years | M | TB | – | – | – | – | – |
| 142 | 1048 | 39 years | M | TB | – | – | – | – | – |
| 143 | 1057 | 35 years | M | Pulmonary TB | – | – | – | – | + |
| 144 | 1072 | 56 years | M | Pulmonary TB | – | – | – | – | – |
| 145 | 1076 | 25 years | F | Pulmonary TB | Pulmonary TB | Pulmonary oedema | – | + | – |
| 146 | 1086 | 45 years | M | Pulmonary TB | – | – | – | – | – |
| 147 | 1093 | 29 years | M | Pulmonary TB | – | – | – | – | – |
| 148 | 1095 | 61 years | M | Pulmonary TB | – | – | – | – | – |
| 149 | 1096R | 40 years | M | Pulmonary TB | – | – | – | – | – |
| 150 | 1105 | 22 years | F | Pulmonary TB | – | – | – | + | + |
| 151 | 1106 | 28 years | M | TB spondylitis, heart disease | TB spondylitis | – | – | – | – |
| 152 | 1113 | 29 years | M | Pulmonary TB | Myocarditis | – | – | – | + |

| No. | Terry No. | Age at death | Sex | Cause of death | | | | Exhibiting ABVIs or not | Exhibiting PAs or not |
|---|---|---|---|---|---|---|---|---|---|
| | | | | MR | DC1 | DC2 | DC3 | | |
| 153 | 1122 | 29 years | F | Pulmonary TB | – | – | – | + | + |
| 154 | 1124R | 49 years | F | Pulmonary TB | – | – | – | – | – |
| 155 | 1129 | 50 years | F | Pulmonary TB | – | – | – | – | – |
| 156 | 1132 | 67 years | M | Pulmonary TB | – | – | – | – | – |
| 157 | 1147R | 32 years | M | Pleural expansion | Possible TB, pleural effusion | – | – | – | – |
| 158 | 1156 | c. 56 years | M | Pulmonary TB | – | – | – | – | – |
| 159 | 1157 | 40 years | M | Pulmonary TB | – | – | – | – | – |
| 160 | 1159 | 26 years | M | Pulmonary TB | – | – | – | – | + |
| 161 | 1165 | c. 26 years | M | Pulmonary TB | – | – | – | + | + |
| 162 | 1169 | 28 years | M | Pulmonary TB | – | – | – | + | + |
| 163 | 1173 | 38 years | F | Pulmonary TB | – | – | – | – | – |
| 164 | 1183 | c. 21 years | M | Pneumonia | Pulmonary TB | Pleurisy | – | – | – |
| 165 | 1185 | 33 years | M | Pulmonary TB | Pulmonary haemorrhage | Pulmonary TB | – | – | – |
| 166 | 1187 | 21 years | M | Pulmonary TB | – | – | – | – | – |

| No. | Terry No. | Age at death | Sex | Cause of death | | | | Exhibiting ABVIs or not | Exhibiting PAs or not |
|---|---|---|---|---|---|---|---|---|---|
| | | | | MR | DC1 | DC2 | DC3 | | |
| 167 | 1190 | c. 64 years | M | Pulmonary TB | – | – | – | – | + |
| 168 | 1205 | 38 years | M | Pulmonary TB | – | – | – | – | – |
| 169 | 1210 | 46 years | F | TB | Pulmonary TB | – | – | – | – |
| 170 | 1215 | 25 years | F | Pulmonary TB | Pulmonary TB | Child birth | – | – | – |
| 171 | 1222 | 28 years | F | Pulmonary TB | – | – | – | + | + |
| 172 | 1226 | c. 48 years | M | Pulmonary TB | – | – | – | – | – |
| 173 | 1230 | 53 years | M | Cysto-urethro-rectocele with ascites | Pulmonary TB | Pneumothorax | Cirrhosis, myocarditis | – | – |
| 174 | 1236 | 36 years | F | TB | – | – | – | – | + |
| 175 | 1247 | 53 years | M | TB | – | – | – | – | – |
| 176 | 1249R | 40 years | F | – | Pulmonary TB | – | – | – | – |
| 177 | 1255 | 39 years | M | TB sinusitis | TB of the hips | *Delirium tremens* | – | – | + |
| 178 | 1263R | 53 years | M | Pulmonary TB | – | – | – | – | – |
| 179 | 1264 | 45 years | M | Pulmonary TB | – | – | – | – | – |
| 180 | 1266R | c. 77 years | M | Senility, lues | Pulmonary TB | Syphilis | Parkinson’s disease, arteriosclerosis | – | – |

| No. | Terry No. | Age at death | Sex | Cause of death | | | | Exhibiting ABVIs or not | Exhibiting PAs or not |
|---|---|---|---|---|---|---|---|---|---|
| | | | | MR | DC1 | DC2 | DC3 | | |
| 181 | 1275 | c. 59 years | M | Pulmonary TB, myocarditis | Pneumonia | Arteriosclerosis | – | – | – |
| 182 | 1278 | 45 years | M | Pulmonary TB | – | – | – | – | – |
| 183 | 1282 | 61 years | M | Myocarditis | Pulmonary TB | Myocarditis | – | – | – |
| 184 | 1285 | 36 years | M | TB | – | – | – | – | + |
| 185 | 1287 | 24 years | F | Pulmonary TB | – | – | – | – | + |
| 186 | 1300 | 28 years | M | TB | – | – | – | – | + |
| 187 | 1304R | 58 years | M | Pulmonary TB | Pulmonary TB | Pericardial effusion | – | – | – |
| 188 | 1309 | 36 years | M | Neurological condition | TB spondylitis | Nephritis | Paralysis of the lower extremities | – | – |
| 189 | 1313 | 63 years | M | TB | Syphilis | Pulmonary TB | Myocarditis, arteriosclerosis | – | + |
| 190 | 1315 | 43 years | M | TB | – | – | – | – | – |
| 191 | 1318 | 43 years | M | Pulmonary TB | – | – | – | – | + |
| 192 | 1319 | c. 36 years | M | Pulmonary TB | Pulmonary TB | Syphilis | – | – | + |
| 193 | 1322 | 34 years | M | Pulmonary TB | – | – | – | + | + |
| 194 | 1331 | 41 years | M | Pulmonary TB | – | – | – | – | – |

| No. | Terry No. | Age at death | Sex | Cause of death | | | | Exhibiting ABVIs or not | Exhibiting PAs or not |
|---|---|---|---|---|---|---|---|---|---|
| | | | | MR | DC1 | DC2 | DC3 | | |
| 195 | 1337RR | 35 years | F | Psychosis | Pulmonary TB | Brain syndrome | – | – | – |
| 196 | 1346 | 73 years | M | Lung abscess | Pulmonary TB | – | – | – | – |
| 197 | 1352 | 28 years | M | Pulmonary TB | – | – | – | – | – |
| 198 | 1359 | 52 years | M | Bronchitis, alcoholism | Pulmonary TB | – | – | – | + |
| 199 | 1362 | 36 years | M | Pulmonary TB | – | – | – | – | – |
| 200 | 1367 | 32 years | M | Pulmonary TB | – | – | – | – | – |
| 201 | 1369 | 52 years | M | Pulmonary TB | – | – | – | + | + |
| 202 | 1377 | c. 52 years | F | Haemorrhage | Haemorrhage into the right ventricle | TB meningitis | – | + | + |
| 203 | 1379 | c. 54 years | M | Myocarditis | Myocarditis | Pulmonary TB | – | – | – |
| 204 | 1388 | 29 years | M | Pulmonary TB | Pulmonary TB | TB peritonitis | Cardiac decompensation | – | + |
| 205 | 1397 | 35 years | M | TB | – | – | – | – | – |
| 206 | 1398 | 31 years | M | TB, meningitis | – | – | – | – | + |
| 207 | 1401 | 47 years | F | Pulmonary TB | – | – | – | – | – |
| 208 | 1406 | 63 years | F | TB | – | – | – | – | – |

| No. | Terry No. | Age at death | Sex | Cause of death | | | | Exhibiting ABVIs or not | Exhibiting PAs or not |
|---|---|---|---|---|---|---|---|---|---|
| | | | | MR | DC1 | DC2 | DC3 | | |
| 209 | 1407 | 44 years | M | TB | – | – | – | – | – |
| 210 | 1419 | 54 years | F | Pulmonary TB | – | – | – | – | – |
| 211 | 1422R | c. 35 years | M | Pulmonary TB | – | – | – | – | – |
| 212 | 1428R | 71 years | M | Pulmonary TB | – | – | – | – | – |
| 213 | 1434R | 19 years | F | Pulmonary TB | – | – | – | – | – |
| 214 | 1451 | 36 years | M | TB | – | – | – | – | – |
| 215 | 1453R | 58 years | M | Pulmonary TB | – | – | – | – | – |
| 216 | 1455 | 68 years | M | TB | TB | Pneumonia | – | – | – |
| 217 | 1458 | 37 years | M | Pulmonary TB | – | – | – | – | + |
| 218 | 1476 | 51 years | F | Pulmonary TB, TB adenitis | – | – | – | – | – |
| 219 | 1503 | 22 years | M | Pulmonary TB | – | – | – | – | – |
| 220 | 1507 | 23 years | F | Pulmonary TB, heart disease | – | – | – | – | – |
| 221 | 1521 | 54 years | M | Pulmonary TB | – | – | – | + | – |
| 222 | 1531 | 40 years | M | Pulmonary TB | – | – | – | – | – |

| No. | Terry No. | Age at death | Sex | Cause of death | | | | Exhibiting ABVIs or not | Exhibiting PAs or not |
|---|---|---|---|---|---|---|---|---|---|
| | | | | MR | DC1 | DC2 | DC3 | | |
| 223 | 1533 | 45 years | M | Pulmonary TB | – | – | – | – | – |
| 224 | 1536 | 71 years | F | Pulmonary TB | – | – | – | – | – |
| 225 | 1539 | 23 years | M | Pulmonary TB | – | – | – | – | – |
| 226 | 1544 | c. 24 years | F | TB | Pulmonary TB | – | – | + | + |
| 227 | 1551 | 25 years | F | ? | Salpingitis | TB peritonitis | – | + | – |
| 228 | 1553 | 30 years | F | Possible TB, heart disease | Nephritis | – | – | – | + |
| 229 | 1555 | 41 years | F | Pulmonary TB | – | – | – | + | – |
| 230 | 1562 | 46 years | F | Pulmonary TB | – | – | – | + | + |
| 231 | 1568 | c. 62 years | F | TB | Pulmonary TB | – | – | – | – |
| 232 | 1572 | c. 44 years | F | Pulmonary TB | – | – | – | – | – |
| 233 | 1576 | 69 years | F | Pulmonary TB | Pulmonary TB | Schizophrenic reaction | – | – | – |
| 234 | 1629 | 80 years | F | Pneumonia | Pulmonary TB | – | – | – | – |
