# Supplementary material for: Tracking down the White Plague. Chapter two: The role of endocranial abnormal blood vessel impressions and periosteal appositions in the paleopathological diagnosis of tuberculous meningitis
Source: PLoS One. 2020 Sep 1;15(9):e0238444. doi: 10.1371/journal.pone.0238444 (PMC7462305; doi:10.1371/journal.pone.0238444)
Supplement: S2 Table — (MR = morgue record; DC1 = death certificate primary; DC2 = death certificate secondary; DC3 = death certificate tertiary; c. = circa; F = female; M = male; TB = tuberculosis; NTB = non-tuberculous; ABVIs = abnormal blood vessel impressions; PAs = periosteal appositions; + = exhibiting ABVIs/PAs;– = not exhibiting ABVIs/PAs). (PDF) [file pone.0238444.s002.pdf]

S2 Table: Basic biographic data of individuals in the NTB group (N=193).
(MR = morgue record; DC1 = death certificate primary; DC2 = death certificate secondary; DC3 = death certificate tertiary; c. = circa; F = female; M = male; TB = tuberculosis; NTB = non-tuberculous; ABVIs = abnormal blood vessel impressions; PAs = periosteal appositions; + = exhibiting ABVIs/PAs; – = not exhibiting ABVIs/PAs)

No.	Terry No.	Age at death	Sex	Cause of death				Exhibiting ABVIs or not	Exhibiting PAs or not
				MR	DC1	DC2	DC3		
1	4R	40 years	M	Antral carcinoma	–	Metastasis of the left orbit	–	–	–
2	12R	46 years	F	Rectal cancer	–	–	–	+	+
3	19R	c. 69 years	F	Hypertensive heart disease	Hypertensive heart disease	–	–	–	–
4	25	c. 65 years	F	–	–	–	–	–	–
5	25R	c. 43 years	F	Alcoholism	Pulmonary oedema	Cerebral oedema	Alcoholism	+	–
6	31R	c. 38 years	M	Stab wound	Cardiac thrombosis	Septic stab wound in the right arm	–	–	–
7	44R	c. 72 years	F	Cerebral vascular disease	Cerebral vascular accident	Hypertensive heart disease	–	–	–
8	46R	c. 67 years	F	Accident	Pneumonia	Subdural haemorrhage from fall	–	–	–
9	47R	78 years	F	Pneumonia	–	–	–	–	–
10	58R	c. 70 years	F	Cardiorenal disease	Hypertensive heart disease	–	–	–	+
11	62RR	c. 38 years	M	–	–	–	–	–	–
12	64R	57 years	F	Cervical cancer	Cancerous cervix with metastasis	–	–	–	–

No.	Terry No.	Age at death	Sex	Cause of death				Exhibiting ABVIs or not	Exhibiting PAs or not
				MR	DC1	DC2	DC3		
13	69	75 years	F	Breast cancer, pneumonia	–	Pneumonia	–	–	–
14	76R	c. 62 years	F	Brain oedema	Myocarditis	Pleural effusion	–	–	–
15	79R	c. 52 years	F	Nephritis	Nephritis	–	–	–	–
16	104RR	c. 80 years	F	–	Cerebral vascular accident	Arteriosclerotic heart disease	–	–	–
17	105R	81 years	F	Heart failure	–	Arteriosclerosis	–	–	–
18	112R	c. 59 years	F	Rectal cancer	–	–	–	–	–
19	124R	61 years	F	Congestive heart failure	Hypertensive heart disease	Nephritis	–	–	–
20	127R	c. 72 years	F	Scleroderma	–	–	–	+	–
21	132R	39 years	F	Heart disease	–	Nephritis	–	–	–
22	134	53 years	F	Pneumonia	Pneumonia	–	–	–	–
23	135R	c. 43 years	F	Heart failure	Coronary thrombosis	–	psychosis, *Dementia precox*	–	–
24	140RR	c. 56 years	F	Pneumonia	–	–	–	+	–
25	141R	c. 83 years	F	Cerebral accident	Arteriosclerotic heart disease	–	–	–	–
26	142R	c. 82 years	F	Pneumonia	–	Fractured hip	Senility	–	–

No.	Terry No.	Age at death	Sex	Cause of death				Exhibiting ABVIs or not	Exhibiting PAs or not
				MR	DC1	DC2	DC3		
27	149R	70 years	F	Nephritis	Uraemia	Arteriosclerosis	Hypertensive heart disease, psychosis	–	–
28	167	77 years	M	Cardiorenal disease	Myocarditis	Lues	–	–	–
29	177R	c. 33 years	F	Haemorrhage	Left lung haemorrhage	–	–	–	–
30	178R	c. 60 years	F	Hypertensive encephalopathy	Cerebral thrombosis	Hypertension	–	–	+
31	179R	c. 76 years	F	Myocarditis	–	–	–	–	–
32	197R	60 years	F	Breast carcinoma	–	–	–	–	–
33	199	c. 70 years	F	Heart failure	Myocarditis	Senility	–	–	–
34	209	38 years	M	Pneumonia	–	–	–	–	–
35	218	56 years	F	*Diabetes mellitus*, asthma	–	–	–	–	–
36	221	c. 47 years	M	Intestinal obstruction	–	–	–	–	–
37	227	57 years	M	Syphilis	–	–	–	–	–
38	231	c. 60 years	M	Syphilis, senility	Breast carcinoma	Syphilis, senility	–	+	–
39	237	70 years	M	Hypopyon ulcer of the eye	–	Pneumonia	–	–	–
40	243R	c. 74 years	F	*Diabetes mellitus*	Myocardial infarction	*Diabetes mellitus*	–	–	–

No.	Terry No.	Age at death	Sex	Cause of death				Exhibiting ABVIs or not	Exhibiting PAs or not
				MR	DC1	DC2	DC3		
41	247R	90 years	F	Myocardial failure	–	–	–	–	–
42	249R	81 years	F	–	Cardiac dilatation	Myocarditis	–	–	–
43	259	52 years	M	Pneumonia	–	–	–	–	–
44	268	62 years	M	Senility	Myocarditis	–	–	–	–
45	272	49 years	F	Cardiac decompensation, nephritis	–	–	–	–	+
46	285	54 years	M	Pneumonia	–	–	–	–	–
47	293R	c. 54 years	F	Coronary occlusion	Coronary sclerosis	–	–	–	–
48	296R	69 years	M	Myocarditis	–	–	–	–	–
49	298	c. 53 years	M	–	–	–	–	–	–
50	306R	c. 50 years	F	Hepatic cirrhosis	Brain oedema	Hepatic cirrhosis	–	–	–
51	314	65 years	M	Senility, pneumonia	–	Myocarditis	–	–	–
52	317	84 years	M	Arteriosclerosis	–	–	–	–	–
53	338	65 years	M	Pneumonia, myocarditis, senility	–	–	–	–	–
54	339R	67 years	F	Myocarditis	–	–	–	–	–

No.	Terry No.	Age at death	Sex	Cause of death				Exhibiting ABVIs or not	Exhibiting PAs or not
				MR	DC1	DC2	DC3		
55	344R	52 years	F	Pneumonia	Nephritis	—	—	—	—
56	347	c. 73 years	M	Syphilis	—	Syphilis	—	—	—
57	348R	c. 28 years	F	Eclampsia	Nephritis	Pregnancy with eclampsia	—	—	—
58	393RR	c. 58 years	F	Accident	Pneumonia	Femur fracture	—	—	—
59	397	60 years	M	Uraemia	Syphilis	Myocarditis	Nephritis	—	—
60	403	52 years	M	Cardiorenal disease	Myocarditis	Nephritis	—	—	—
61	422	c. 20 years	M	Pneumonia	—	—	—	—	—
62	437R	c. 44 years	F	Pneumonia	—	—	—	—	—
63	438	c. 86 years	M	—	—	—	—	—	—
64	445	66 years	M	Ruptured gastric ulcer, peritonitis	—	—	—	—	—
65	447	49 years	M	—	Nephritis	—	—	—	—
66	452	c. 61 years	M	Syphilis, senility	Syphilis	Nephritis	—	—	—
67	453	c. 42 years	M	Gangrenous bowel	Strangulated inguinal hernia	—	—	—	—
68	458	41 years	M	Haemorrhoids	Nephritis	—	—	—	—

No.	Terry No.	Age at death	Sex	Cause of death				Exhibiting ABVIs or not	Exhibiting PAs or not
				MR	DC1	DC2	DC3		
69	463	c. 50 years	M	Myocarditis, rheumatism	–	–	–	–	–
70	465	25 years	M	Post-operative wound of the chest	Aortic aneurysm	–	–	–	–
71	470	c. 44 years	M	Cardiorenal disease	–	–	–	–	+
72	477	22 years	M	Paralysis of the lower extremities	Toxaemia from bed sores	Septicaemia from ulcers of the buttocks	–	+	–
73	483	44 years	M	Pneumonia	–	–	–	–	–
74	496	54 years	M	Pellagra	–	–	–	–	–
75	497	c. 72 years	M	Nephritis	–	–	–	–	–
76	506	48 years	M	Myocarditis	Asthma	Myocarditis	–	–	–
77	512	c. 50 years	F	Mental condition	Myocarditis	Pneumonia	–	+	–
78	513RR	c. 38 years	F	–	Haemorrhagic pachyonychia	–	–	–	–
79	527	c. 54 years	M	Intestinal carcinoma	Diarrhoea	Enteritis	Myocarditis	–	–
80	528	c. 64 years	F	Cerebral haemorrhage	Myocarditis	–	–	–	–
81	534	c. 50 years	M	Stenosis	–	–	–	+	–
82	536	60 years	M	Cerebral haemorrhage	–	Arteriosclerosis	–	–	+

No.	Terry No.	Age at death	Sex	Cause of death				Exhibiting ABVIs or not	Exhibiting PAs or not
				MR	DC1	DC2	DC3		
83	545	77 years	M	Heart disease	Myocarditis	Arteriosclerosis	–	–	–
84	552	72 years	M	Myocarditis	Nephritis	Myocarditis	–	–	–
85	573	59 years	M	Syphilis	–	–	–	–	–
86	582	c. 49 years	M	Pneumonia	–	–	–	–	–
87	586	40 years	F	Cardiorenal disease	Nephritis	–	–	–	–
88	597	c. 61 years	M	Myocarditis	Valvular heart disease	–	–	–	–
89	602	49 years	M	Pleurisy	Influenza	Pulmonary infarct & abscess	Pulmonary haemorrhage	–	–
90	608	45 years	M	Pneumonia, mitral regurgitation	Myocarditis	Nephritis	–	–	–
91	617R	43 years	F	Pneumonia	–	Encephalic malacia	–	–	+
92	627R	c. 27 years	F	Poisoning	Arsenical poisoning	Neosalvarsan	–	–	–
93	629	c. 72 years	M	Pneumonia, senility	–	Hemiplegia	–	–	+
94	636	51 years	M	Cardiac condition	Myocarditis	Bronchitis	–	–	–
95	657R	c. 28 years	F	Mitral stenosis	–	–	–	–	–
96	686	c. 78 years	F	Myocarditis, senile dementia	–	–	–	–	+

No.	Terry No.	Age at death	Sex	Cause of death				Exhibiting ABVIs or not	Exhibiting PAs or not
				MR	DC1	DC2	DC3		
97	694	c. 52 years	M	Myocarditis, arteriosclerosis	Myocarditis	Arteriosclerosis	–	–	–
98	702R	47 years	M	Myocardial insufficiency	Hypertensive heart disease	Nephritis	–	–	–
99	726	c. 62 years	M	Myocarditis, syphilis	–	–	–	–	–
100	727	c. 45 years	M	Gastric carcinoma	–	–	–	–	–
101	759	60 years	M	Cardiorenal disease	Myocarditis	–	–	–	+
102	789	40 years	M	Syphilis	–	–	–	–	–
103	795	c. 57 years	M	Carcinoma of the cervical glands	–	–	–	–	–
104	809R	60 years	M	Hypertensive heart disease	–	–	–	–	–
105	823	c. 60 years	M	Cerebral apoplexy	–	–	–	–	–
106	833R	47 years	M	Blastomycosis	–	–	–	–	–
107	834R	87 years	F	Degenerative heart disease	Arteriosclerosis	–	–	–	–
108	863	73 years	M	Senility	Cardiorenal disease	Pneumonia	–	–	–
109	891	39 years	F	Pneumonia	–	–	–	–	–
110	903R	c. 51 years	F	–	Subdural brain haemorrhage from fall	–	–	–	–

No.	Terry No.	Age at death	Sex	Cause of death				Exhibiting ABVIs or not	Exhibiting PAs or not
				MR	DC1	DC2	DC3		
111	919	c. 55 years	M	Myocarditis	–	–	–	–	–
112	930R	c. 80 years	F	Cerebral apoplexy	–	–	–	–	–
113	934	62 years	F	Myocarditis, heart failure	–	–	–	–	–
114	938	73 years	M	Myocarditis, sigmoid cancer	Pneumonia	Myocarditis	–	–	–
115	941	59 years	M	Cardiac condition	Myocarditis	–	–	–	+
116	946	48 years	M	Pneumonia	–	–	–	–	+
117	948	39 years	F	Heart disease	Myocarditis	–	–	–	+
118	957R	76 years	F	Femur fracture	–	–	–	–	–
119	964	49 years	M	Cerebral apoplexy	Cerebral haemorrhage	–	–	–	+
120	968	48 years	M	Neurological condition	Lues	–	–	–	–
121	973	66 years	M	Pneumonia	Myocarditis	Pneumonia	–	–	–
122	1023	c. 20 years	M	Car accident	–	–	–	–	–
123	1029R	30 years	M	Parkinson’s disease	–	–	–	–	–
124	1045	c. 41 years	M	Nephritis	Pulmonary embolism	Myocarditis	Nephritis	–	–

No.	Terry No.	Age at death	Sex	Cause of death				Exhibiting ABVIs or not	Exhibiting PAs or not
				MR	DC1	DC2	DC3		
125	1046	c. 60 years	M	–	Hypertensive heart disease	–	–	–	–
126	1050	64 years	M	Intestinal stasis	Intestinal obstruction	Peritonitis	–	–	–
127	1058	68 years	F	Myocarditis	Cerebral apoplexy	Cerebral haemorrhage	Myocarditis	–	–
128	1060	c. 65 years	M	Asthma, myocarditis	–	–	–	–	–
129	1066R	62 years	M	Pneumonia	Degenerative heart disease	Arteriosclerosis	–	+	–
130	1070	c. 51 years	M	Nephritis	Myocarditis	Nephritis	–	–	–
131	1071R	c. 70 years	F	Accident	Endocarditis	Stenosis, hip fracture	–	–	–
132	1098	c. 60 years	M	Cerebral haemorrhage	Cerebral haemorrhage	Arteriosclerosis	–	–	–
133	1100RR	c. 76 years	F	Brain syndrome	Arteriosclerotic heart disease	Brain syndrome	–	–	–
134	1102R	c. 76 years	F	Coronary thrombosis	Coronary thrombosis	Arteriosclerotic heart disease	–	–	–
135	1107	78 years	M	Myocarditis, senility	–	–	–	–	–
136	1130R	79 years	M	Convulsions	Cerebral haemorrhage	Arteriosclerosis	–	–	–
137	1133RR	c. 80 years	F	Arteriosclerosis, avitaminosis, dehydration	Malnutrition	Senility	–	–	–
138	1134R	70 years	F	Hyperarthritis	Arteriosclerosis	Senile psychosis	Hyperarthritis	–	–

No.	Terry No.	Age at death	Sex	Cause of death				Exhibiting ABVIs or not	Exhibiting PAs or not
				MR	DC1	DC2	DC3		
139	1137R	c. 78 years	F	Pneumonia	Degenerative heart disease	Pneumonia	Arteriosclerosis	–	–
140	1138R	37 years	M	Pneumonia	Pneumonia	Pleural effusion	–	–	–
141	1140	46 years	M	Cardiac decompensation	Myocarditis	–	–	–	–
142	1163	41 years	F	Pellagra	–	–	–	–	–
143	1182R	c. 48 years	F	Pneumonia	Aortitis	–	–	–	–
144	1186	c. 44 years	F	Lung abscess	Lung abscess	Pneumonia	–	–	–
145	1192	58 years	M	Pneumonia	–	–	–	–	–
146	1204R	74 years	M	Heart disease	Arteriosclerotic heart disease	–	–	+	–
147	1219	79 years	M	Myocarditis, nephritis	Myocarditis	Senility	–	–	–
148	1224	60 years	M	Mediastinal tumour	Aortic aneurysm	–	–	–	+
149	1228	79 years	M	Nephritis, pneumonia	Myocarditis	Pneumonia	–	–	–
150	1229	c. 73 years	M	Gangrenous foot	Gangrenous foot	Arteriosclerosis	Myocarditis	–	–
151	1232	57 years	M	Pneumonia, myocarditis, arteriosclerosis	–	–	–	–	–
152	1243R	c. 49 years	F	Myocarditis	–	–	–	+	+

No.	Terry No.	Age at death	Sex	Cause of death				Exhibiting ABVIs or not	Exhibiting PAs or not
				MR	DC1	DC2	DC3		
153	1252	50 years	F	Uterine fibroid	Nephritis	Uraemia	–	–	–
154	1267	c. 78 years	M	Myocarditis, senility	–	–	–	–	–
155	1271	58 years	M	Lues	Lues	Myocarditic	Cardiac decompensation	+	–
156	1277	c. 59 years	M	Heat stroke	Epilepsy	–	–	–	–
157	1291	c. 52 years	M	Cardiac decompensation	Hypertensive heart disease	–	–	–	–
158	1299R	52 years	M	Gastric ulcer	Gastric ulcer	Lues	–	–	–
159	1310	c. 85 years	M	Cardiovascular reactivity	Arteriosclerotic heart disease	Auricular fibrillation	–	–	–
160	1342	c. 74 years	M	Hypertensive heart disease	–	–	–	–	–
161	1343	c. 73 years	F	Heart failure, nephritis	Myocarditis	Nephritis	Hypertension	–	–
162	1347R	38 years	F	Cardiorenal disease	Hypertensive heart disease	–	–	–	–
163	1353R	c. 50 years	F	Pneumonia	–	–	–	–	–
164	1368	37 years	M	Appendicitis	Gangrenous appendix	Pneumonia	–	–	+
165	1375R	c. 38 years	M	Pneumonia	–	–	–	–	–
166	1376	46 years	M	Pneumonia	Pneumonia	Myocarditis	–	–	–

No.	Terry No.	Age at death	Sex	Cause of death				Exhibiting ABVIs or not	Exhibiting PAs or not
				MR	DC1	DC2	DC3		
167	1378	54 years	M	Peritonitis	–	–	–	–	–
168	1387	40 years	M	Lues	–	–	–	–	+
169	1405	75 years	F	Myocarditis	–	–	–	–	–
170	1411R	65 years	M	–	Nephritis, uraemia	Hypertrophied prostate	–	–	–
171	1416	c. 70 years	F	Hypertensive heart disease	Arteriosclerotic heart disease	Senile psychosis	–	–	–
172	1417R	37 years	F	Dysentery	Perianal & ischiorectal abscesses	Staphylococcosis	Secondary anaemia	–	–
173	1435	66 years	F	Myocarditis, arteriosclerosis	–	–	–	–	–
174	1439R	56 years	M	Epilepsy, hypertension	Pneumonia	Arteriosclerotic dementia	–	–	–
175	1444	59 years	M	Heart disease	Arteriosclerotic heart disease	–	–	–	–
176	1467	73 years	M	Gastric malignancy	Arteriosclerotic heart disease	Cardiac decompensation	–	–	–
177	1495	89 years	M	Myocarditis	Myocarditis	Pneumonia	–	–	–
178	1502R	c. 82 years	F	Arteriosclerosis	Arteriosclerosis	Senility	–	–	–
179	1505R	41 years	F	Hemiplegia	Hypertension	Cerebral haemorrhage	–	–	–
180	1514	c. 70 years	M	Hemiplegia, strictures	–	–	–	–	–

No.	Terry No.	Age at death	Sex	Cause of death				Exhibiting ABVIs or not	Exhibiting PAs or not
				MR	DC1	DC2	DC3		
181	1519	71 years	M	Myocarditis	Myocarditis, cardiac decompensation	–	–	–	+
182	1534	44 years	M	Urinary bladder calculi	Pyelonephritis, catarrhal cystitis	Bladder & urethral calculi	–	–	–
183	1543	c. 70 years	M	Thrombosis	–	–	–	–	–
184	1549	c. 40 years	F	Cerebral apoplexy	–	–	–	–	–
185	1552	84 years	F	Hypertension, senility	Cardiac decompensation	Arteriosclerotic vascular disease	–	–	–
186	1554	c. 56 years	F	Hypertensive heart disease	–	–	–	–	–
187	1567	c. 69 years	F	Stroke, heart failure	–	–	–	–	–
188	1581	64 years	F	Arteriosclerotic heart disease	–	–	–	–	–
189	1592	53 years	F	Arteriosclerotic heart disease	–	–	–	–	–
190	1599	c. 41 years	F	Suicide	–	–	–	–	–
191	1604	50 years	F	Cerebral haemorrhage	Cerebral haemorrhage	Hypertensive vascular disease	–	–	+
192	1614	38 years	F	–	Congestive heart failure	Rheumatic mitral valve disease	Mental deficiency	–	–
193	1627	c. 90 years	F	Hypertensive heart disease, failure	Arteriosclerotic heart disease	–	–	–	–
