# Supplementary material for: Tracking down the White Plague. Chapter two: The role of endocranial abnormal blood vessel impressions and periosteal appositions in the paleopathological diagnosis of tuberculous meningitis
Source: PLoS One. 2020 Sep 1;15(9):e0238444. doi: 10.1371/journal.pone.0238444 (PMC7462305; doi:10.1371/journal.pone.0238444)
Supplement: S3 Table. Distribution of individuals exhibiting ABVIs in the Terry Collection by affected cranial bones (considering the left and right greater wings of the sphenoid bone as two separate bones), extent, and number of lesions (TB = tuberculosis; NTB = non-tuberculous; ABVIs = abnormal blood vessel i — (PDF) [file pone.0238444.s003.pdf]

S3 Table: Distribution of individuals exhibiting ABVIs in the Terry Collection by affected cranial bones (considering the left and right greater wings of the sphenoid bone as two separate bones), extent, and number of lesions (TB = tuberculosis; NTB = non-tuberculous; ABVIs = abnormal blood vessel impressions; L = left; R = right). Number of individuals in the A) TB group and B) NTB group.

A) TB group (Σ=234)		Frontal bone	Parietal bone (L)	Parietal bone (R)	Temporal bone (L)	Temporal bone (R)	Sphenoid bone (L)	Sphenoid bone (R)	Occipital bone
		42/50 (84.00%)	38/50 (76.00%)	36/50 (72.00%)	0/50 (0.00%)	2/50 (4.00%)	1/50 (2.00%)	1/50 (2.00%)	16/50 (32.00%)
Extent (x) of lesions	x < 25%	13/42 (30.95%)	27/38 (71.05%)	23/36 (63.89%)	–	1/2 (50.00%)	–	–	15/16 (93.75%)
	25% ≤ x < 50%	16/42 (38.10%)	4/38 (10.53%)	6/36 (16.67%)	–	–	–	–	1/16 (6.25%)
	50% ≤ x < 75%	7/42 (16.67%)	5/38 (13.16%)	5/36 (13.89%)	–	1/2 (50.00%)	1/1 (100.00%)	–	–
	75% ≤ x	6/42 (14.29%)	2/38 (5.26%)	2/36 (5.56%)	–	–	–	1/1 (100.00%)	–
Number of lesions	Unifocal	–	2/38 (5.26%)	2/36 (5.56%)	–	2/2 (100.00%)	1/1 (100.00%)	1/1 (100.00%)	3/16 (18.75%)
	Multifocal	42/42 (100.00%)	36/38 (94.74%)	34/36 (94.44%)	–	–	–	–	13/16 (81.25%)

B) NTB group (Σ=193)		Frontal bone	Parietal bone (L)	Parietal bone (R)	Temporal bone (L)	Temporal bone (R)	Sphenoid bone (L)	Sphenoid bone (R)	Occipital bone
		5/12 (41.67%)	9/12 (75.00%)	10/12 (83.33%)	0/12 (0.00%)	0/12 (0.00%)	0/12 (0.00%)	0/12 (0.00%)	1/12 (8.33%)
Extent (x) of lesions	x < 25%	2/5 (40.00%)	6/9 (66.67%)	7/10 (70.00%)	–	–	–	–	1/1 (100.00%)
	25% ≤ x < 50%	2/5 (40.00%)	2/9 (22.22%)	2/10 (20.00%)	–	–	–	–	–
	50% ≤ x < 75%	1/5 (20.00%)	1/9 (11.11%)	1/10 (10.00%)	–	–	–	–	–
	75% ≤ x	–	–	–	–	–	–	–	–
Number of lesions	Unifocal	–	1/9 (11.11%)	1/10 (10.00%)	–	–	–	–	–
	Multifocal	5/5 (100.00%)	8/9 (88.89%)	9/10 (90.00%)	–	–	–	–	1/1 (100.00%)
