# Supplementary material for: Tracking down the White Plague. Chapter two: The role of endocranial abnormal blood vessel impressions and periosteal appositions in the paleopathological diagnosis of tuberculous meningitis
Source: PLoS One. 2020 Sep 1;15(9):e0238444. doi: 10.1371/journal.pone.0238444 (PMC7462305; doi:10.1371/journal.pone.0238444)
Supplement: S5 Table — (TB = tuberculosis; TBM = tuberculous meningitis; ABVIs = abnormal blood vessel impressions; APDIs = abnormally pronounced digital impressions; PAs = periosteal appositions; GIs = granular impressions; + = present; − = not present). (PDF) [file pone.0238444.s005.pdf]

S5 Table: Individual data of cases exhibiting ABVIs regarding other probable TBM-associated endocranial bony changes in the TB group (Σ=50). (TB = tuberculosis; TBM = tuberculous meningitis; ABVIs = abnormal blood vessel impressions; APDIs = abnormally pronounced digital impressions; PAs = periosteal appositions; GIs = granular impressions; + = present; − = not present)

No.	Terry No.	ABVIs	APDIs	PAs	GIs
1	13R	+	+	−	−
2	23R	+	−	−	−
3	84	+	+	−	+
4	95	+	+	−	+
5	128	+	+	+	−
6	145R	+	−	−	+
7	158R	+	−	−	+
8	204	+	+	+	−
9	220	+	+	−	+
10	235	+	+	−	−
11	254	+	+	+	−
12	255	+	+	−	+
13	265	+	+	−	−
14	279	+	+	−	+
15	280	+	+	+	+
16	304	+	+	+	−
17	306	+	+	+	−
18	329	+	−	−	+
19	353	+	−	−	−
20	358R	+	−	−	−
21	522	+	+	+	+
22	568	+	−	+	+
23	571	+	−	−	−
24	621R	+	+	+	−
25	739	+	−	−	+
26	822	+	+	−	+
27	896RR	+	+	−	+
28	902	+	−	−	−
29	932	+	+	+	−
30	933R	+	+	−	+
31	955	+	+	+	−
32	987	+	+	+	+
33	1020	+	+	−	+
34	1027	+	−	+	+
35	1030	+	−	−	−
36	1033	+	+	+	−

No.	Terry No.	ABVIs	APDIs	PAs	GIs
37	1076	+	+	−	+
38	1105	+	+	+	−
39	1122	+	+	+	−
40	1165	+	+	+	−
41	1169	+	+	+	−
42	1222	+	+	+	+
43	1322	+	+	+	−
44	1369	+	+	+	−
45	1377	+	−	+	−
46	1521	+	+	−	−
47	1544	+	−	+	−
48	1551	+	+	−	−
49	1555	+	+	−	−
50	1562	+	+	+	−
