# Supplementary material for: Tracking down the White Plague. Chapter two: The role of endocranial abnormal blood vessel impressions and periosteal appositions in the paleopathological diagnosis of tuberculous meningitis
Source: PLoS One. 2020 Sep 1;15(9):e0238444. doi: 10.1371/journal.pone.0238444 (PMC7462305; doi:10.1371/journal.pone.0238444)
Supplement: S6 Table — (TB = tuberculosis; TBM = tuberculous meningitis; PAs = periosteal appositions; APDIs = abnormally pronounced digital impressions; ABVIs = abnormal blood vessel impressions; GIs = granular impressions; + = present; − = not present). (PDF) [file pone.0238444.s006.pdf]

S6 Table: Individual data of cases exhibiting PAs regarding other probable TBM-associated endocranial bony changes in the TB group (Σ=47). (TB = tuberculosis; TBM = tuberculous meningitis; PAs = periosteal appositions; APDIs = abnormally pronounced digital impressions; ABVIs = abnormal blood vessel impressions; GIs = granular impressions; + = present; − = not present)

No.	Terry No.	PAs	APDIs	ABVIs	GIs
1	128	+	+	+	−
2	204	+	+	+	−
3	254	+	+	+	−
4	280	+	+	+	+
5	304	+	+	+	−
6	306	+	+	+	−
7	385	+	+	−	−
8	423	+	+	−	−
9	522	+	+	+	+
10	568	+	−	+	+
11	621R	+	+	+	−
12	846	+	+	−	−
13	897	+	+	−	−
14	915	+	+	−	−
15	932	+	+	+	−
16	955	+	+	+	−
17	987	+	+	+	+
18	1027	+	−	+	+
19	1033	+	+	+	−
20	1034	+	+	−	−
21	1057	+	+	−	+
22	1105	+	+	+	−
23	1113	+	+	−	−
24	1122	+	+	+	−
25	1159	+	+	−	+
26	1165	+	+	+	−
27	1169	+	+	+	−
28	1190	+	+	−	−
29	1222	+	+	+	+
30	1236	+	+	−	−
31	1255	+	+	−	−
32	1285	+	+	−	−
33	1287	+	+	−	−
34	1300	+	+	−	−
35	1313	+	+	−	−
36	1318	+	+	−	+

No.	Terry No.	PAs	APDIs	ABVIs	GIs
37	1319	+	+	−	+
38	1322	+	+	+	−
39	1359	+	+	−	−
40	1369	+	+	+	−
41	1377	+	−	+	−
42	1388	+	+	−	+
43	1398	+	+	−	−
44	1458	+	+	−	+
45	1544	+	−	+	−
46	1553	+	+	−	−
47	1562	+	+	+	−
