# Supplementary material for: Tracking down the White Plague. Chapter two: The role of endocranial abnormal blood vessel impressions and periosteal appositions in the paleopathological diagnosis of tuberculous meningitis
Source: PLoS One. 2020 Sep 1;15(9):e0238444. doi: 10.1371/journal.pone.0238444 (PMC7462305; doi:10.1371/journal.pone.0238444)
Supplement: S7 Table — (TB = tuberculosis; ABVIs = abnormal blood vessel impressions; PNBFs = periosteal new bone formations; HPO = hypertrophic pulmonary osteopathy; + = present; − = not present). (PDF) [file pone.0238444.s007.pdf]

S7 Table: Individual data of cases exhibiting ABVIs on the inner skull surface regarding possible TB-related non-endocranial bony changes in the TB group (Σ=50).
(TB = tuberculosis; ABVIs = abnormal blood vessel impressions; PNBFs = periosteal new bone formations; HPO = hypertrophic pulmonary osteopathy;
+ = present; − = not present)

No.	Terry No.	ABVIs	PNBFs on the visceral costal surfaces	HPO	Extra-spinal osteomyelitis	Extra-spinal arthritis	Vertebral hypervascularization	Vertebral lytic lesions and/or arthritis	Reactive new bone formations indicative of a cold abscess
1	13R	+	+	−	−	−	+	−	−
2	23R	+	+	−	−	−	−	−	−
3	84	+	−	−	−	−	−	+	+
4	95	+	−	+	−	−	−	+	+
5	128	+	+	−	−	−	+	−	−
6	145R	+	+	−	−	−	+	−	−
7	158R	+	+	+	−	−	−	−	−
8	204	+	+	−	−	+	+	−	−
9	220	+	−	−	−	−	−	−	−
10	235	+	−	−	−	−	+	−	+
11	254	+	+	+	−	−	−	−	−
12	255	+	+	−	−	−	+	−	−

No.	Terry No.	ABVIs	PNBFs on the visceral costal surfaces	HPO	Extra-spinal osteomyelitis	Extra-spinal arthritis	Vertebral hypervascularization	Vertebral lytic lesions and/or arthritis	Reactive new bone formations indicative of a cold abscess
13	265	+	+	−	−	−	+	+	+
14	279	+	+	−	−	−	+	+	−
15	280	+	−	−	−	−	+	−	−
16	304	+	−	−	−	−	+	−	−
17	306	+	+	+	−	−	+	−	−
18	329	+	+	−	+	−	−	+	+
19	353	+	−	−	−	−	+	−	+
20	358R	+	+	−	−	−	+	−	−
21	522	+	+	−	−	−	−	−	−
22	568	+	+	−	−	−	+	−	−
23	571	+	−	−	−	−	+	−	−
24	621R	+	+	−	−	−	+	−	−
25	739	+	−	−	−	−	+	−	−
26	822	+	+	−	−	−	+	−	−

No.	Terry No.	ABVIs	PNBFs on the visceral costal surfaces	HPO	Extra-spinal osteomyelitis	Extra-spinal arthritis	Vertebral hypervascularization	Vertebral lytic lesions and/or arthritis	Reactive new bone formations indicative of a cold abscess
27	896RR	+	+	–	–	+	+	–	–
28	902	+	+	+	+	+	+	+	+
29	932	+	–	–	–	–	–	–	–
30	933R	+	+	–	–	–	+	–	–
31	955	+	+	+	–	–	+	–	–
32	987	+	+	+	–	–	–	–	–
33	1020	+	+	+	–	–	+	–	–
34	1027	+	–	–	–	–	–	–	–
35	1030	+	–	–	–	–	–	–	–
36	1033	+	+	–	–	–	+	–	–
37	1076	+	+	–	–	–	+	–	–
38	1105	+	+	+	–	–	+	–	–
39	1122	+	+	+	–	–	–	–	–
40	1165	+	–	–	–	–	+	–	–

No.	Terry No.	ABVIs	PNBFs on the visceral costal surfaces	HPO	Extra-spinal osteomyelitis	Extra-spinal arthritis	Vertebral hypervascularization	Vertebral lytic lesions and/or arthritis	Reactive new bone formations indicative of a cold abscess
41	1169	+	+	−	−	−	+	−	−
42	1222	+	−	−	−	−	−	−	−
43	1322	+	+	−	−	−	+	−	−
44	1369	+	+	−	−	−	+	−	−
45	1377	+	−	−	−	−	+	−	−
46	1521	+	+	−	−	−	−	−	−
47	1544	+	+	−	−	−	+	−	−
48	1551	+	−	−	−	−	+	−	−
49	1555	+	+	+	−	−	−	−	−
50	1562	+	−	−	−	−	−	−	−
