# Supplementary material for: Tracking down the White Plague. Chapter two: The role of endocranial abnormal blood vessel impressions and periosteal appositions in the paleopathological diagnosis of tuberculous meningitis
Source: PLoS One. 2020 Sep 1;15(9):e0238444. doi: 10.1371/journal.pone.0238444 (PMC7462305; doi:10.1371/journal.pone.0238444)
Supplement: S8 Table — (TB = tuberculosis; PAs = periosteal appositions; PNBFs = periosteal new bone formations; HPO = hypertrophic pulmonary osteopathy; + = present; − = not present). (PDF) [file pone.0238444.s008.pdf]

S8 Table: Individual data of cases exhibiting PAs on the inner skull surface regarding possible TB-related non-endocranial bony changes in the TB group (Σ=47).
(TB = tuberculosis; PAs = periosteal appositions; PNBFs = periosteal new bone formations; HPO = hypertrophic pulmonary osteopathy;
+ = present; − = not present)

No.	Terry No.	PAs	PNBFs on the visceral costal surfaces	HPO	Extra-spinal osteomyelitis	Extra-spinal arthritis	Vertebral hypervascularization	Vertebral lytic lesions and/or arthritis	Reactive new bone formations indicative of a cold abscess
1	128	+	+	−	−	−	+	−	−
2	204	+	+	−	−	+	+	−	−
3	254	+	+	+	−	−	−	−	−
4	280	+	−	−	−	−	+	−	−
5	304	+	−	−	−	−	+	−	−
6	306	+	+	+	−	−	+	−	−
7	385	+	−	−	−	−	+	−	−
8	423	+	+	−	−	−	+	−	−
9	522	+	+	−	−	−	−	−	−
10	568	+	+	−	−	−	+	−	−
11	621R	+	+	−	−	−	+	−	−
12	846	+	+	−	−	−	+	−	−

No.	Terry No.	PAs	PNBFs on the visceral costal surfaces	HPO	Extra-spinal osteomyelitis	Extra-spinal arthritis	Vertebral hypervascularization	Vertebral lytic lesions and/or arthritis	Reactive new bone formations indicative of a cold abscess
13	897	+	+	−	−	−	+	+	−
14	915	+	+	−	−	−	+	−	−
15	932	+	−	−	−	−	−	−	−
16	955	+	+	+	−	−	+	−	−
17	987	+	+	+	−	−	−	−	−
18	1027	+	−	−	−	−	−	−	−
19	1033	+	+	−	−	−	+	−	−
20	1034	+	−	−	−	−	−	−	−
21	1057	+	+	−	−	−	−	−	−
22	1105	+	+	+	−	−	+	−	−
23	1113	+	−	−	−	−	−	−	−
24	1122	+	+	+	−	−	−	−	−
25	1159	+	+	+	−	−	+	−	−
26	1165	+	−	−	−	−	+	−	−

No.	Terry No.	PAs	PNBFs on the visceral costal surfaces	HPO	Extra-spinal osteomyelitis	Extra-spinal arthritis	Vertebral hypervascularization	Vertebral lytic lesions and/or arthritis	Reactive new bone formations indicative of a cold abscess
27	1169	+	+	–	–	–	+	–	–
28	1190	+	+	–	–	–	–	–	–
29	1222	+	–	–	–	–	–	–	–
30	1236	+	–	–	–	–	+	–	–
31	1255	+	–	–	–	–	–	+	+
32	1285	+	–	–	+	+	–	+	–
33	1287	+	+	–	–	–	+	+	+
34	1300	+	+	–	–	–	+	–	–
35	1313	+	–	–	–	–	–	–	–
36	1318	+	+	–	–	–	–	–	–
37	1319	+	+	–	–	–	+	–	–
38	1322	+	+	–	–	–	+	–	–
39	1359	+	–	–	–	–	–	–	–
40	1369	+	+	–	–	–	+	–	–

No.	Terry No.	PAs	PNBFs on the visceral costal surfaces	HPO	Extra-spinal osteomyelitis	Extra-spinal arthritis	Vertebral hypervascularization	Vertebral lytic lesions and/or arthritis	Reactive new bone formations indicative of a cold abscess
41	1377	+	−	−	−	−	+	−	−
42	1388	+	+	−	−	−	+	−	−
43	1398	+	+	−	−	−	+	−	−
44	1458	+	+	−	−	−	−	−	−
45	1544	+	+	−	−	−	+	−	−
46	1553	+	−	−	−	−	+	−	−
47	1562	+	−	−	−	−	−	−	−
