# Supplementary material for: Tracking down the White Plague. Chapter two: The role of endocranial abnormal blood vessel impressions and periosteal appositions in the paleopathological diagnosis of tuberculous meningitis
Source: PLoS One. 2020 Sep 1;15(9):e0238444. doi: 10.1371/journal.pone.0238444 (PMC7462305; doi:10.1371/journal.pone.0238444)
Supplement: S9 Table — (NTB = non-tuberculous; TBM = tuberculous meningitis; ABVIs = abnormal blood vessel impressions; APDIs = abnormally pronounced digital impressions; PAs = periosteal appositions; GIs = granular impressions; + = present; − = not present). (PDF) [file pone.0238444.s009.pdf]

S9 Table: Individual data of cases exhibiting ABVIs regarding other probable TBM-associated endocranial bony changes in the NTB group (Σ=12). (NTB = non-tuberculous; TBM = tuberculous meningitis; ABVIs = abnormal blood vessel impressions; APDIs = abnormally pronounced digital impressions; PAs = periosteal appositions; GIs = granular impressions; + = present; − = not present)

No.	Terry No.	ABVIs	APDIs	PAs	GIs
1	12R	+	−	+	−
2	25R	+	−	−	−
3	127R	+	+	−	−
4	140RR	+	−	−	−
5	231	+	+	−	−
6	477	+	−	−	−
7	512	+	+	−	−
8	534	+	−	−	−
9	1066R	+	−	−	−
10	1204R	+	−	−	−
11	1243R	+	−	+	−
12	1271	+	−	−	−
