# Supplementary material for: Tracking down the White Plague. Chapter two: The role of endocranial abnormal blood vessel impressions and periosteal appositions in the paleopathological diagnosis of tuberculous meningitis
Source: PLoS One. 2020 Sep 1;15(9):e0238444. doi: 10.1371/journal.pone.0238444 (PMC7462305; doi:10.1371/journal.pone.0238444)
Supplement: S10 Table — (NTB = non-tuberculous; TBM = tuberculous meningitis; PAs = periosteal appositions; APDIs = abnormally pronounced digital impressions; ABVIs = abnormal blood vessel impressions; GIs = granular impressions; + = present; − = not present). (PDF) [file pone.0238444.s010.pdf]

S10 Table: Individual data of cases exhibiting PAs regarding other probable TBM-associated endocranial bony changes in the NTB group (Σ=20). (NTB = non-tuberculous; TBM = tuberculous meningitis; PAs = periosteal appositions; APDIs = abnormally pronounced digital impressions; ABVIs = abnormal blood vessel impressions; GIs = granular impressions; + = present; − = not present)

No.	Terry No.	PAs	APDIs	ABVIs	GIs
1	12R	+	−	+	−
2	58R	+	+	−	−
3	178R	+	−	−	−
4	272	+	+	−	+
5	470	+	+	−	−
6	536	+	+	−	−
7	617R	+	−	−	−
8	629	+	−	−	−
9	686	+	−	−	−
10	759	+	+	−	−
11	941	+	−	−	−
12	946	+	+	−	−
13	948	+	−	−	−
14	964	+	−	−	−
15	1224	+	+	−	−
16	1243R	+	−	+	−
17	1368	+	−	−	−
18	1387	+	−	−	−
19	1519	+	−	−	−
20	1604	+	−	−	−
