# Supplementary material for: Tracking down the White Plague. Chapter two: The role of endocranial abnormal blood vessel impressions and periosteal appositions in the paleopathological diagnosis of tuberculous meningitis
Source: PLoS One. 2020 Sep 1;15(9):e0238444. doi: 10.1371/journal.pone.0238444 (PMC7462305; doi:10.1371/journal.pone.0238444)
Supplement: S12 Table — (NTB = non-tuberculous; PAs = periosteal appositions; PNBFs = periosteal new bone formations; HPO = hypertrophic pulmonary osteopathy; + = present; − = not present). (PDF) [file pone.0238444.s012.pdf]

S12 Table: Individual data of cases exhibiting PAs on the inner skull surface regarding possible TB-related non-endocranial bony changes in the NTB group (Σ=20).
(NTB = non-tuberculous; PAs = periosteal appositions; PNBFs = periosteal new bone formations; HPO = hypertrophic pulmonary osteopathy;
+ = present; − = not present)

No.	Terry No.	PAs	PNBFs on the visceral costal surfaces	HPO	Extra-spinal osteomyelitis	Extra-spinal arthritis	Vertebral hypervascularization	Vertebral lytic lesions and/or arthritis	Reactive new bone formations indicative of a cold abscess
1	12R	+	−	−	−	−	−	−	−
2	58R	+	−	−	−	−	−	−	−
3	178R	+	−	−	−	−	−	−	−
4	272	+	−	−	−	−	−	−	−
5	470	+	−	−	−	−	−	−	−
6	536	+	−	−	−	−	−	−	−
7	617R	+	−	−	−	−	−	−	−
8	629	+	−	−	−	−	−	−	−
9	686	+	−	−	−	−	−	−	−
10	759	+	−	−	−	−	−	−	−
11	941	+	−	−	−	−	−	−	−
12	946	+	−	−	−	−	+	−	−

No.	Terry No.	PAs	PNBFs on the visceral costal surfaces	HPO	Extra-spinal osteomyelitis	Extra-spinal arthritis	Vertebral hypervascularization	Vertebral lytic lesions and/or arthritis	Reactive new bone formations indicative of a cold abscess
13	948	+	+	–	–	–	–	–	–
14	964	+	–	–	–	–	–	–	–
15	1224	+	–	–	–	–	–	–	–
16	1243R	+	–	–	–	–	–	–	–
17	1368	+	–	–	–	–	–	–	–
18	1387	+	–	–	–	–	–	–	–
19	1519	+	–	–	–	–	–	–	–
20	1604	+	–	–	–	–	–	–	–
