# Supplementary material for: Tracking down the White Plague. Chapter two: The role of endocranial abnormal blood vessel impressions and periosteal appositions in the paleopathological diagnosis of tuberculous meningitis
Source: PLoS One. 2020 Sep 1;15(9):e0238444. doi: 10.1371/journal.pone.0238444 (PMC7462305; doi:10.1371/journal.pone.0238444)
Supplement: Attachment — Submitted filename: response_to_reviewers_Spekker_et_al.doc [file pone.0238444.s013.doc]

Dr. JJ Cray Jr., PhD

Academic Editor

PLOS ONE

August 13, 2020

Dear Dr. JJ Cray Jr.,

I am very thankful for the reviewer’s insightful and constructive comments regarding our manuscript entitled “Tracking down the White Plague. Chapter two: The role of endocranial abnormal blood vessel impressions and periosteal appositions in the paleopathological diagnosis of tuberculous meningitis” that was submitted to PLOS ONE (manuscript ID: PONE-D-20-18111). I am sure that the reviewer helped us to improve the quality of our manuscript. The main text has been modified following the reviewer’s suggestions, and the revised version of our manuscript has been uploaded to the submission site of PLOS ONE.

**Responses to the suggestions:**

1) **Reviewer 1** mentioned that individuals from the NTB group who died of accidents could be analysed as a subgroup, and they might represent a better “control” group for the specific goals of our paper. Following **Reviewer 1**’s request, we pulled out the aforementioned individuals from the NTB group. They can be found in the table below:

| **No.** | **Terry No.** | **Cause of death** | | | | **Exhibiting ABVIs or not** | **Exhibiting PAs or not** |
| --- | --- | --- | --- | --- | --- | --- | --- |
|  |  | **MR** | **DC1** | **DC2** | **DC3** |  |  |
| **1** | **46R** | **Accident** | **Pneumonia** | **Subdural haemorrhage from fall** | **–** | **–** | **–** |
| **2** | **393RR** | **Accident** | **Pneumonia** | **Femur fracture** | **–** | **–** | **–** |
| **3** | **1023** | **Car accident** | **–** | **–** | **–** | **–** | **–** |
| **4** | **1071R** | **Accident** | **Endocarditis** | **Stenosis, hip fracture** | **–** | **–** | **–** |
| **5** | **142R** | **Pneumonia** | **–** | **Fractured hip** | **Senility** | **–** | **–** |
| **6** | **903R** | **–** | **Subdural haemorrhage from fall** | **–** | **–** | **–** | **–** |
| **7** | **957R** | **Femur fracture** | **–** | **–** | **–** | **–** | **–** |

In the original NTB group, the morgue record and/or death certificate stated an accident as the cause of death in four cases (Terry No. 46R, 393RR, 1023, and 1071R). In three additional cases (Terry No.142R, 903R, and 957R), fall, and hip or femur fracture were recorded as the cause of death – these could also be considered as accidents. Unfortunately, as **Reviewer 1** already predicted, the number of individuals in the “NTB-accident” group (n=7) would be too low compared to the number of individuals in the TB group (n=234) and in the NTB group without these seven individuals (n=186) for appropriate statistical analysis. Nevertheless, as it can be seen in the table above, none of the seven individuals died of an accident exhibited ABVIs or PAs on the inner surface of the skull. On the other hand, we agree with **Reviewer 1** that individuals died of an accident could still have some underlying TB infection or NTB disease associated with meningeal infection. Nevertheless, this is also true for individuals died of NTB disease not associated with meningeal infection. Therefore, we think that very likely, the “NTB-accident” group would not represent a better “control” group for the goals of our paper.

2) **Reviewer 1** asked if any of the individuals in the Terry Collection were specifically identified as having died of meningococcal disease (*Neisseria meningitidis* infection) or other diseases associated with meningeal infections. Following **Reviewer 1**’s request, we checked all the individuals, and found five cases with the aforementioned conditions as the recorded cause of death: Terry No. 734RR (60-year-old, female) – meningococcal meningitis, Terry No. 455 (25-year-old, female) and Terry No. 719 (25-year-old, male) – luetic meningitis, Terry No. 530 (57-year-old, female) – streptococcal meningitis, and Terry No. 1284R (62-year-old, male) – pneumococcal meningitis. Unfortunately, none of these individuals were included into the NTB group; therefore, they were not evaluated regarding the presence of ABVIs and PAs. (The individuals composing the NTB group were selected randomly by using a random number generator.)

3) As for lines 228–231, **Reviewer 1** asked us to give in parentheses the number of individuals with ABVIs dying of cardiovascular problems, respiratory diseases, syphilis, etc. We agree with **Reviewer 1** that it would be easier for the readers if they would not have to go to the supplementary table to find this information, and we modified the main text accordingly.

“In the NTB group, the most frequently registered NTB causes of death were cardiovascular problems (six cases), followed by respiratory diseases (three cases), syphilis (two cases), and different types of cancer (two cases) among individuals exhibiting ABVIs on the inner surface of the skull – in several cases, more than one of these conditions were recorded as the cause of death (S2 Table).”

4) **Reviewer 1** noted that in the figures, it would be helpful if we would add small arrows to draw the reader’s attention specifically to the features we wish to highlight. We agree with **Reviewer 1** and added the requested arrows in Figures 1, 3, and 5.

In the revised version of our manuscript, we tried to execute all suggestions of the reviewer. I hope this new version will be suitable for publication in PLOS ONE.

Thank you again for the reviewer’s insightful and constructive comments and your editorial work!

Sincerely yours,

Dr. Olga Spekker, PhD

Postdoctoral researcher

Department of Biological Anthropology, University of Szeged

Közép fasor 52, H-6726 Szeged, Hungary

Email: [olga.spekker@gmail.com](mailto:olga.spekker@gmail.com)

Tel: +36 20 807 72 94
